# High Circulating Follicle-Stimulating Hormone Level Is a Potential Risk Factor for Renal Dysfunction in Post-Menopausal Women

**DOI:** 10.3389/fendo.2021.627903

**Published:** 2021-04-01

**Authors:** Qihang Li, Dongmei Zheng, Haiyan Lin, Fang Zhong, Jing Liu, Yafei Wu, Zhixiang Wang, Qingbo Guan, Meng Zhao, Ling Gao, Jiajun Zhao

**Affiliations:** ^1^Department of Endocrinology, Shandong Provincial Hospital, Cheeloo College of Medicine, Shandong University, Jinan, China; ^2^Shandong Clinical Medical Center of Endocrinology and Metabolism, Shandong Academy of Clinical Medicine, Jinan, China; ^3^Institute of Endocrinology and Metabolism, Shandong Academy of Clinical Medicine, Jinan, China; ^4^Department of Endocrinology, Shandong Provincial Hospital Affiliated to Shandong First Medical University, Jinan, China; ^5^Health Management Center, Shandong Provincial Hospital Affiliated to Shandong First Medical University, Jinan, China; ^6^Department of Scientific Center, Shandong Provincial Hospital Affiliated to Shandong First Medical University, Jinan, China

**Keywords:** FSH, menopause, eGFR, CKD, renal dysfunction, aging

## Abstract

**Objective:**

Menopause contributes to renal dysfunction in women, which is generally attributed to estrogen withdrawal. In addition to decreased estrogen level, serum follicle-stimulating hormone (FSH) level increases after menopause. This study investigated the association between high circulating FSH level and renal function in post-menopausal women.

**Methods:**

This observational cross-sectional study included 624 pre-menopausal, 121 peri-menopausal, and 2540 post-menopausal women. The levels of female sex hormones were examined by chemiluminescence and indices of renal function were measured using a clinical chemistry analyzer. The post-menopausal women were grouped into quartiles according to serum FSH levels.

**Results:**

Renal function progressively declined from pre-menopause to peri-menopause to post-menopause, which was accompanied by increasing serum FSH level. In post-menopausal women, serum creatinine level increased with increasing FSH quartile, which was accompanied by a decrease in estimated glomerular filtration rate (eGFR) (p for trend <0.001); moreover, the prevalence of declined eGFR (<90 ml/min/1.73 m^2^) and chronic kidney disease (CKD; eGFR <60 ml/min/1.73 m^2^) increased (p for trend <0.001). Even after adjusting for confounders, the odds ratios (ORs) of declined eGFR and CKD increased with increasing FSH quartiles in post-menopausal women. The ORs of declined eGFR (OR=2.19, 95% confidence interval [CI]: 1.63–2.92) and CKD (OR=10.09, 95% CI: 2.28–44.65) in the highest FSH quartile were approximately 2- and 10-fold higher, respectively, than in the lowest FSH quartile (p<0.05). After stratifying post-menopausal women by median age (61 years), the OR for declined eGFR for each FSH quartile in the older group was higher than that for the corresponding FSH quartile in the younger group.

**Conclusions:**

A high circulating FSH level is an independent risk factor for renal dysfunction in women after menopause. Additionally, aging may aggravate the association of high FSH levels with reduced renal function in post-menopausal women.

## Introduction

Chronic kidney disease (CKD) is a major public health problem, with a global prevalence estimated at 5–10%. The burden of CKD-associated diseases is alarmingly high, which is primarily due to cardiovascular morbidity and mortality (1). Kidney function is influenced by sex hormones. Earlier researching findings suggested that female sex hormones were overall protective against the progression of kidney disease (2–4); however, a meta-analysis of 11 studies revealed a more rapid decline in kidney function in women (who were mostly post-menopausal) compared to men (5). Moreover, the renoprotection associated with female sex hormones appears to diminish after menopause (6), and epidemiologic studies have reported a substantial increase in CKD incidence in women after menopause (7, 8). A recent study in the United States reported that women—mostly post-menopausal—accounted for 42% of patients on dialysis (9). These findings suggest that the changes in sex hormone levels associated with menopause have adverse effects on kidney function.

Estrogen attenuates glomerulosclerosis and tubulointerstitial fibrosis; thus, the decline in estrogen level after menopause can promote the progression of CKD (10–12). The occurrence of CKD was found to be lower in women who received oral hormone replacement therapy (HRT) than in those who did not receive the treatment (13). However, estrogen supplementation did not completely reverse the decline in renal function in post-menopausal women (14), indicating that factors other than estrogen withdrawal contribute to the observed decline in renal function following menopause.

Menopause is characterized by the absence of menstrual cycles as a result of ovarian failure and decreased circulating estrogen concentrations along with a compensatory increase in circulating follicle-stimulating hormone (FSH) (8, 10–12). FSH acts on non-gonadal tissues through the FSH receptor. We previously reported that FSH affects hepatic cholesterol biosynthesis, hepatic gluconeogenesis, and osteoarthritis (15–17). In the present study, we investigated the association between FSH levels and renal function in women in the context of menopause; we also examined whether FSH is an independent risk factor for renal dysfunction in post-menopausal women, and the influence of age on this association.

## Materials and Methods

### Study Design and Participants

**Figure 1:**
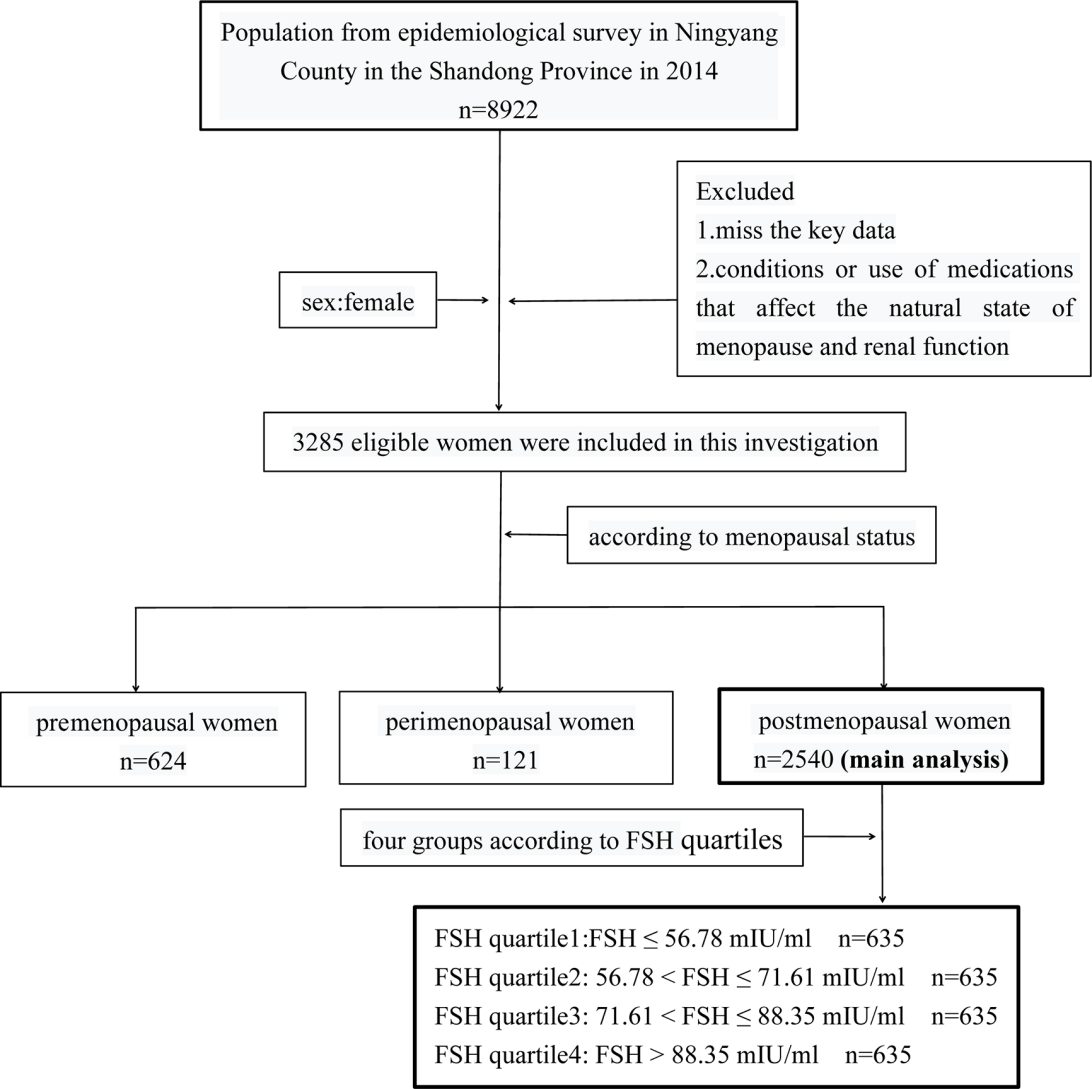
Flow chart for selection or grouping.

This cross-sectional study was conducted in Ningyang County, Shandong Province, China in 2014 as part of the Risk Evaluation of cAncers in Chinese diabeTic Individuals: A lONgitudinal (REACTION) trial at Shanghai Jiao Tong University School of Medicine (clinical trial number: NCT01506869). The study was approved by Ruijin Hospital Ethics Committee of Shanghai JiaoTong University School of Medicine. Informed consent was obtained from each participant after a detailed explanation of the purpose and nature of all procedures used. Of the 8922 participants in the REACTION trial, 3285 eligible women were included in the present study who met the following criteria: 1) available key data such as age, body mass index (BMI), renal function indices, sex hormone levels, serum lipid profiles, menstrual history, hypertension, diabetes, etc.; 2) no condition affecting the natural state of menopause and renal function such as premature ovarian failure, bilateral ovariectomy, uterectomy, pregnancy, lactation, chronic nephritis, nephrotic syndrome, nephrectomy, and malignant tumors; and 3) no use of medications affecting the natural state of menopause and renal function including estrogens, androgens, progesterone, glucocorticoids, thiazide diuretics, methoxyflurane, tetracycline, penicillin, and sulphonamides. The women were assigned to one of three groups according to menopausal status—ie, pre-menopause (n=624), peri-menopause (n=121), and post-menopause (n=2540) (**Figure 1**).

### Data Collection

All investigators completed a standardized training program to minimize inter-rater variability. For pre-menopausal women, venipuncture was scheduled on days 2–5 of a regular menstrual cycle to exclude periodic differences in sex hormone levels; for peri- and post-menopausal women, venipuncture was performed at random times (18).

All blood samples were collected between 8:00 a.m. and 10:00 a.m. after overnight fasting and immediately preserved at −80°C until use. Blood glucose level was measured within 2 h. Female sex hormone levels were evaluated by chemiluminescence (Cobas E601; Roche, Basel, Switzerland). The sensitivity for FSH detection was 0.100 mIU/ml, and the range of measurement was 0.100–200.0 mIU/ml; for E2, the sensitivity and range of measurement was 5 pg/ml and 5–3000 pg/ml respectively. Intra- and inter-assay coefficients of variation were always <5% for FSH and E2. Serum lipid profiles, plasma glucose levels, and indices of hepatic and renal functions were examined using a Beckman AU5800 chemistry analyzer (Beckman Coulter, Tokyo, Japan). Blood pressure was measured three times at 3-min intervals for each participant using an electronic sphygmomanometer (HEM-7117; Omron, Kyoto, Japan), and the mean value was calculated. Height (cm) and weight (kg) were recorded and used to calculate BMI (kg/m^2^).

### Definitions of Study Outcomes

Menopausal status was determined based on responses to a self-report questionnaire regarding menstrual history or amenorrhea. Pre-menopause was defined as the presence of menses within the past 3 months. Peri-menopause was defined as the presence of menses within the past 3 months with menstrual irregularity in the year preceding the questionnaire, or 3–11 months of amenorrhea. We selected subjects with 3–11 months of amenorrhea as peri-menopause because their E2 levels were similar to those in post-menopause. Post-menopause was defined as the cessation of menstruation for a minimum of 12 months (17, 19). Renal dysfunction was defined as declined estimated glomerular filtration rate (eGFR<90 ml/min/1.73 m^2^) or CKD (eGFR<60 ml/min/1.73m^2^). Dyslipidaemia was defined as follows: 1) high total cholesterol (≥ 6.22 mmol/l); 2) high triglyceride (≥1.70 mmol/l); 3) high low-density lipoprotein cholesterol (≥ 4.14mmol/l); 4) high free fatty acids (≥ 0.9 mmol/l); and 5) low high-density lipoprotein cholesterol <1.30 mmol/l) (20). Hypertension and diabetes were diagnosed based on self-reported previous diagnosis, or were defined as systolic blood pressure ≥130 mmHg or diastolic blood pressure ≥ 85 mmHg for hypertension (21) and fasting plasma glucose ≥7.0 mmol/l or post-prandial 2-h plasma glucose ≥11.1 mmol/l for diabetes (we chooe the former). Never smoking or drinking was assigned a value of 0; otherwise, it was assigned a value of 1.

### Statistical Analysis

Statistical analyses were performed using SPSS v24.0 for Windows (IBM Corp, Armonk, NY, USA). Continuous variables with normal and skewed distributions are presented as mean ± standard deviation and median with interquartile range, respectively. Categorical variables are presented as numbers and percentages. Comparisons of various indices among the three menopausal states or among FSH quartiles in post-menopausal women were performed by one-way analysis of variance (ANOVA) for continuous variables with normal distribution; with the Kruskal–Wallis test for continuous variables with skewed distribution; and with the chi-squared test for categorical variables. ANOVA and Cochran–Armitage chi-squared test were used to assess the trends in continuous and categorical variables, respectively, in the transitions across menopausal states or increasing FSH quartiles in post-menopausal women. A linear regression model was used to evaluate the relationship between FSH levels and eGFR levels in post-menopausal women. A multivariate logistic regression model was used to evaluate the association of FSH quartiles with the prevalence of renal dysfunction in post-menopausal women with adjustments for age, oestradiol (E2), BMI, dyslipidaemia, hypertension, diabetes, smoking, and drinking. The outcomes of the logistic regression analysis are presented as regression coefficient (B), adjusted odds ratio (OR), and 95% confidence interval (CI). All calculated p values were two-sided, and p<0.05 was considered statistically significant.

## Results

### Sex Hormone Levels and Renal Function Indices in Pre-, Peri-, and Post-Menopausal Women

The 3285 women included in the study were divided into pre-menopause (n=624), peri-menopause (n=121), and post-menopause (n=2540) groups. The clinical characteristics of the groups are summarized in **Table 1**. With the change in menopausal status from pre-menopause to peri- and post-menopause, median serum E2 level decreased sharply from 66.75 to 19.12 to 5.58 pg/ml, while median serum FSH level increased from 8.65 to 56.49 to 71.50 mIU/ml. Age, which is an irreversible risk factor for renal dysfunction (22), increased significantly across the three groups from 43.06 to 51.36 to 60.94 years (p for trend <0.001). The prevalence of decreased eGFR increased from pre-menopause to peri- and post-menopause (6.1% to 14.9% to 49.0%; p for trend <0.001), with a corresponding increase in prevalence of CKD (p for trend=0.001). Additionally, in post-menopausal women, mean serum creatinine (Scr) increased from 57.47 to 64.55 μmol/l and mean eGFR level decreased from 108.27 to 88.73 ml/min/1.73 m^2^ (both p<0.001 vs pre-menopausal women).

**Table 1:** Clinical characteristics of the three groups according to menopausal status.

	Pre-menopause (n=624)	Peri-menopause (n=121)	Post-menopause (n=2540)	P value	P for trend
Age (years)	43.06 ± 8.37	51.36 ± 4.41^a^	60.94 ± 7.07^a,b^	<0.001	<0.001
BMI (kg/m^2^)	25.38 ± 3.83	26.33 ± 4.21^a^	25.62 ± 3.85	0.046	0.178
FSH (mIU/mL)	8.65 (6.06-16.67)	56.49 (32.67-79.15)^a^	71.50 (56.63-88.16)^a,b^	<0.001	<0.001
E2 (pg/mL)	66.75 (32.46-137.70)	19.12 (8.38-89.63)^a^	5.58 (5.00-12.81)^a,b^	<0.001	<0.001
LH (mIU/mL)	6.87 (4.72-14.79)	32.91 (22.77-44.47)^a^	31.34 (24.61-39.80)^a^	<0.001	<0.001
Scr (μml/L)	57.47 ± 8.26	61.50 ± 6.53^a^	64.55 ± 9.70^a,b^	<0.001	<0.001
eGFR (ml/min/1.73 m^2^)	108.27 ± 12.70	98.03 ± 8.30^a^	88.73 ± 11.30^a,b^	<0.001	<0.001
Declined eGFR, n (%)	38 (6.1%)	18 (14.9%)	1245 (49.0%)	<0.001	<0.001
CKD, n (%)	1 (0.2%)	0 (0.0%)	47 (1.9%)	0.003	0.001
UA (μml/L)	266 (226-309)	285 (242-339)^a^	290 (242-342)^a^	<0.001	<0.001
TG (mmol/L)	0.99 (0.76-1.40)	1.09 (0.79-1.67)	1.22 (0.90-1.76)^a^	<0.001	<0.001
TC (mmol/L)	4.93 ± 0.91	5.41 ± 0.92^a^	5.70 ± 1.03^a,b^	<0.001	<0.001
LDL-C (mmol/L)	2.71 ± 0.72	3.09 ± 0.75^a^	3.32 ± 0.83^a,b^	<0.001	<0.001
HDL (mmol/L)	1.35 (1.15-1.56)	1.42 (1.19-1.71)^a^	1.44 (1.25-1.66)^a^	<0.001	<0.001
FFA (mmol/L)	0.56 (0.42-0.72)	0.63 (0.49-0.79)^a^	0.67 (0.51-0.86)^a,b^	<0.001	<0.001
AST (U/L)	20 (17-23)	21 (17-27)^a^	20 (18-24)	0.014	0.017
ALT (U/L)	14 (11-18)	17 (12-24)	16 (13-21)	<0.001	<0.001
SBP (mmHg)	121 ± 20	140 ± 28^a^	141 ± 22^a^	<0.001	<0.001
DBP (mmHg)	73 ± 14	78 ± 14	80 ± 12^a^	<0.001	<0.001
FPG (mmol/L)	5.3 (5.0-5.7)	5.5 (5.1-5.9)	5.8 (5.3-6.5)^a^	<0.001	<0.001
Hypertension, n (%)	78 (12.5%)	38 (31.7%)	874 (34.7%)	<0.001	<0.001
Diabetes, n (%)	37 (6.0%)	10 (8.3%)	419 (16.5%)	<0.001	<0.001
Smoking, n (%)	5 (0.9%)	0 (0.0%)	44 (2.0%)	0.070	0.043
Drinking, n (%)	39 (6.7%)	6 (5.7%)	263 (11.8%)	<0.001	<0.001

All data are expressed as mean ± standard deviation, median (interquartile range), number (percentage), and significance (P value and P for trend). BMI, body mass index; FSH, follicle-stimulating hormone; E2, oestradiol; Scr, serum creatinine; UA, uric acid; eGFR, estimated glomerular filtration rate; declined eGFR, eGFR<90 ml/min/1.73 m^2^; CKD, chronic kidney diseases; TG, triglyceride; TC, total cholesterol; LDL-C, low-density lipoprotein cholesterol; H-LDL, high density lipoprotein cholesterol; FFA, free fatty acid; AST, aspartate aminotransferase; ALT, alanine aminotransferase; SBP, systolic blood pressure; DBP, diastolic blood pressure; FPG, fasting plasma glucose.

^a^compared with pre-menopausal women (P < 0.05).

^b^compared with peri-menopausal women (P < 0.05).

### Renal Function Declines With Increasing FSH Level in Post-Menopausal Women

**Figure 2:**
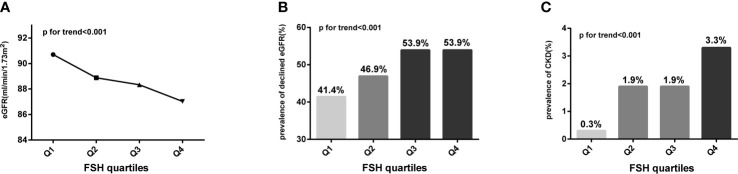
Trends for various indicators of renal function with increasing FSH quartiles (expressed as Q1, Q2, Q3, and Q4) in post-menopausal women. **(A)** Trend for eGFR; **(B)** trend for the prevalence of declined eGFR; **(C)** trend for the prevalence of CKD. Analysis of variance (ANOVA) trend test and Cochran–Armitage chi-squared tests for trend were conducted to assess the trends of continuous and categorical variables, respectively. FSH, follicle-stimulating hormone; eGFR, estimated glomerular filtration rate; declined eGFR, eGFR<90 mL/min/1.73 m^2^; CKD, chronic kidney disease.

We divided post-menopausal women into quartiles according to serum FSH level (mIU/ml) as follows: quartile 1: FSH ≤ 56.78 mIU/ml, quartile 2: 56.78<FSH ≤ 71.61 mIU/ml, quartile 3: 71.61<FSH ≤ 88.35 mIU/ml, and quartile 4: FSH>88.35 mIU/ml. With increasing FSH quartile, mean Scr level increased from 62.98 to 66.55 μmol/l (p for trend <0.001) whereas eGFR level decreased from 90.70 to 87.03 ml/min/1.73 m^2^ (p for trend <0.001). The *post hoc* test showed that mean Scr or eGFR levels between any two FSH quartiles differed significantly (p<0.05), except between quartiles 2 and 3 (p=0.408 and 0.388, respectively). Similarly, the prevalence of declined eGFR increased from 41.4% to 53.9% (p for trend <0.001) while that of CKD increased from 0.3% to 3.3% (p for trend <0.001) with increasing FSH quartiles (**Figure 2** and **Table 2**). On the other hand, age did not show any trend across FSH quartiles (p for trend=0.316) and E2 levels were extremely low in all quartiles. Linear regression analysis showed that FSH levels were negatively associated with eGFR levels in post-menopausal women even after adjustment for potential confounders, such as age, years since menopause, LH, E2, BMI, lipid profiles (including TC, TG, LDL-C, LDL-C, HDL-C, FFA), diabetes, hypertension, smoking, drinking (Beta=-0.128 in model 1; Beta=-0.125 in model 2; Beta=-0.172 in model 3; all p values <0.001), (**Table 3**). These results indicate that FSH has negative relationship with renal function in post-menopausal women.

**Table 2:** Comparisons among groups according to FSH quartiles in postmenopausal women.

FSH level (mIU/mL) by quartile	Quartile 1 (~56.78)	Quartile 2 (56.78–71.61)	Quartile 3 (71.61–88.35)	Quartile 4 (88.35~)	P value	P for trend
Age (years)	60.21 ± 7.28	61.53 ± 6.57^a^	61.34 ± 6.89^a^	60.69 ± 7.44^b^	0.003	0.316
Menopausal age (years)	49.79 ± 4.48	49.52 ± 3.53	48.00 ± 4.00	48.30 ± 4.59	0.207	0.056
Years since menopause (years)	11 (5-18)	19 (12-27)	19 (12-27)	19 (11-26)	0.334	0.127
BMI (kg/m^2^)	27.01 ± 3.97	25.96 ± 3.62^a^	25.29 ± 3.76^a,b^	24.21 ± 3.48^a,b,c^	<0.001	<0.001
E2 (pg/mL)	8.19 (5.00–17.26)	5.62 (5.00–13.06)^a^	5.78 (5.00–11.77)^a^	5.00 (5.00–9.41)^a,b^	<0.001	<0.001
LH (mIU/mL)	22.38 (17.54–26.87)	28.85 (24.65–33.48)^a^	33.82 (29.16–39.20)^a,b^	44.74 (37.90–51.59)^a,b,c^	<0.001	<0.001
eGFR (ml/min/1.73 m^2^)	90.70 ± 9.90	88.88 ± 11.32^a^	88.33 ± 10.17^a^	87.03 ± 13.22^a,b,c^	<0.001	<0.001
Declined eGFR, n (%)	263 (41.4%)	298 (46.9%)	342 (53.9%)	342 (53.9%)	<0.001	<0.001
CKD, n (%)	2 (0.3%)	12 (1.9%)	12 (1.9%)	21 (3.3%)	0.001	<0.001
Scr (μml/L)	62.98 ± 7.11	64.12 ± 9.65^a^	64.56 ± 7.48^a^	66.55 ± 13.06^a,b,c^	<0.001	<0.001
UA (μml/L)	305 ± 78	301 ± 77	294 ± 72	293 ± 76	0.012	0.001
TG (mmol/L)	1.38 (0.97–1.97)	1.22 (0.91–1.72)^a^	1.21 (0.90–1.73)^a^	1.10 (0.83–1.61)^a,b^	<0.001	<0.001
TC (mmol/L)	5.58 ± 1.00	5.69 ± 0.99	5.74 ± 1.05^a^	5.78 ± 1.06^a^	<0.001	<0.001
LDL-C (mmol/L)	3.25 ± 0.82	3.35 ± 0.82	3.38 ± 0.86^a^	3.31 ± 0.84	0.045	0.158
HDL-C (mmol/L)	1.36 (1.18–1.60)	1.45 (1.24–1.63)^a^	1.44 (1.25–1.66)^a^	1.53 (1.34–1.77)^a,b,c^	<0.001	<0.001
FFA (mmol/L)	0.68 (0.55–0.85)	0.68 (0.53–0.89)	0.65 (0.51–0.86)	0.66 (0.49–0.85)	0.186	0.086
AST (U/L)	20 (17–24)	21 (17–25)	20 (18–24)	21 (18–25)	0.005	0.003
ALT (U/L)	16 (13–21)	16 (13–21)	15 (12–21)	15 (13–20)	0.108	0.252
SBP (mmHg)	144 ± 25	139 ± 19	138 ± 20	141 ± 22	0.404	0.472
DBP (mmHg)	81 ± 12	79 ± 10	81 ± 13	79 ± 14	0.428	0.199
FPG (mmol/L)	6.54 ± 2.06	6.65 ± 2.26	6.40 ± 2.20^b^	6.09 ± 1.84^a,b,c^	<0.001	<0.001
hypertension, n (%)	240 (38.0%)	218 (34.8%)	222 (35.4%)	194 (30.8%)	0.061	0.014
diabetes, n (%)	126 (19.8%)	108 (17.1%)	105 (16.6%)	80 (12.6%)	0.007	0.001
Smoking, n (%)	10 (1.8%)	14 (2.5%)	12 (2.2%)	8 (1.4%)	0.632	0.614
Drinking, n (%)	70 (12.5%)	64 (11.5%)	72 (13.0%)	57 (10.1%)	0.460	0.359

All data are expressed as mean ± standard deviation, median (interquartile range), number (percentage), and significance (P value and P for trend). BMI, body mass index; FSH, follicle-stimulating hormone; E2, oestradiol; Scr, serum creatinine; UA, uric acid; eGFR, estimated glomerular filtration rate; declined eGFR, eGFR<90 mL/min/1.73 m^2^; CKD, chronic kidney diseases; TG, triglyceride; TC, total cholesterol; LDL-C, low-density lipoprotein cholesterol; H-LDL, high density lipoprotein cholesterol; FFA, free fatty acid; AST, aspartate aminotransferase; ALT, alanine aminotransferase; SBP, systolic blood pressure; DBP, diastolic blood pressure; FPG, fasting plasma glucose.

^a^compared with FSH quartile1 (P < 0.05).

^b^compared with FSH quartile2 (P < 0.05).

^c^compared with FSH quartile3 (P < 0.05).

**Table 3:** Linear regression analysis of FSH levels and eGFR levels in post-menopausal women.

	B	SE	95% CI of B	Beta	P value
Univariable model	-0.059	0.008	-0.076 to -0.043	-0.138	<0.001
Multivariable model 1	-0.054	0.007	-0.067 to -0.041	-0.128	<0.001
Multivariable model 2	-0.053	0.007	-0.066 to -0.039	-0.125	<0.001
Multivariable model 3	-0.072	0.008	-0.087 to -0.057	-0.172	<0.001

Data are unstandardized coefficients (B), corresponding standard error (SE), 95% confidence interval (CI) of B, standardized coefficients (Beta), and significance (p value).

Multivariable model 1 was adjusted for age, years since menopause.

Multivariable model 2 was further adjusted for LH, E2.

Multivariable model 3 was further adjusted for BMI, lipid profiles (including TC, TG, LDL-C, LDL-C, HDL-C, FFA), diabetes, hypertension, smoking, drinking.

BMI, body mass index; FSH, follicle-stimulating hormone; LH, luteinizing hormone; E2, oestradiol; eGFR, estimated glomerular filtration rate; TG, triglyceride; TC, total cholesterol; LDL-C, low-density lipoprotein cholesterol; H-LDL, high density lipoprotein cholesterol; FFA, free fatty acid.

### FSH is An Independent Risk Factor for Renal Dysfunction in Post-Menopausal Women

To investigate whether FSH contributes to renal dysfunction in post-menopausal women, we conducted a multivariate logistic stepwise regression analysis. After adjusting for potential confounders including age, years since menopause, LH, E2, BMI, dyslipidaemia, diabetes, hypertension, smoking, and drinking, the ORs of declined eGFR and CKD were two-fold (OR=2.187, 95% CI: 1.635–2.924) and 10-fold (OR=10.088, 95% CI: 2.279–44.650) higher, respectively, in the highest FSH quartile compared to the lowest (p<0.05), (**Table 4**).

**Table 4:** Multivariate stepwise logistic regression of FSH quartiles for the presence of renal dysfunction in postmenopausal women.

	B	S.E.	OR (95% CI)	P value
Declined eGFR				
FSH quartile 1			1 (ref)	
FSH quartile 2	0.113	0.144	1.120 (0.844-1.485)	0.432
FSH quartile 3	0.653	0.147	1.922 (1.441-2.563)	<0.001
FSH quartile 4	0.782	0.148	2.187 (1.635-2.924)	<0.001
CKD				
FSH quartile 1			1 (ref)	
FSH quartile 2	1.407	0.801	4.082 (0.849-19.615)	0.079
FSH quartile 3	1.509	0.793	4.523 (0.956-21.391)	0.057
FSH quartile 4	2.311	0.759	10.088 (2.279-44.650)	0.002

Dependent variable: declined eGFR or CKD; independent variable: FSH quartiles; data are expressed as coefficient (B), standard error (S.E.), adjusted odds ratio (OR), 95% confidence interval (CI), and significance (P value). Multivariate model: adjusted for age, years since menopause, LH, E2, BMI, dyslipidaemia (yes=1, no=0): high TC, high TG, high LDL-C, high LDL-C, high FFA, low HDL-C; diabetes (yes=1, no=0), hypertension (yes=1, no=0), smoking (yes=1, no=0), drinking (yes=1, no=0).

FSH, follicle-stimulating hormone; LH, luteinizing hormone; eGFR, estimated glomerular filtration rate; declined eGFR, eGFR<90 ml/min/1.73 m^2^; CKD, chronic kidney disease.

### Age Influences the Association Between FSH and Renal Dysfunction in Post-Menopausal Women

**Figure 3:**
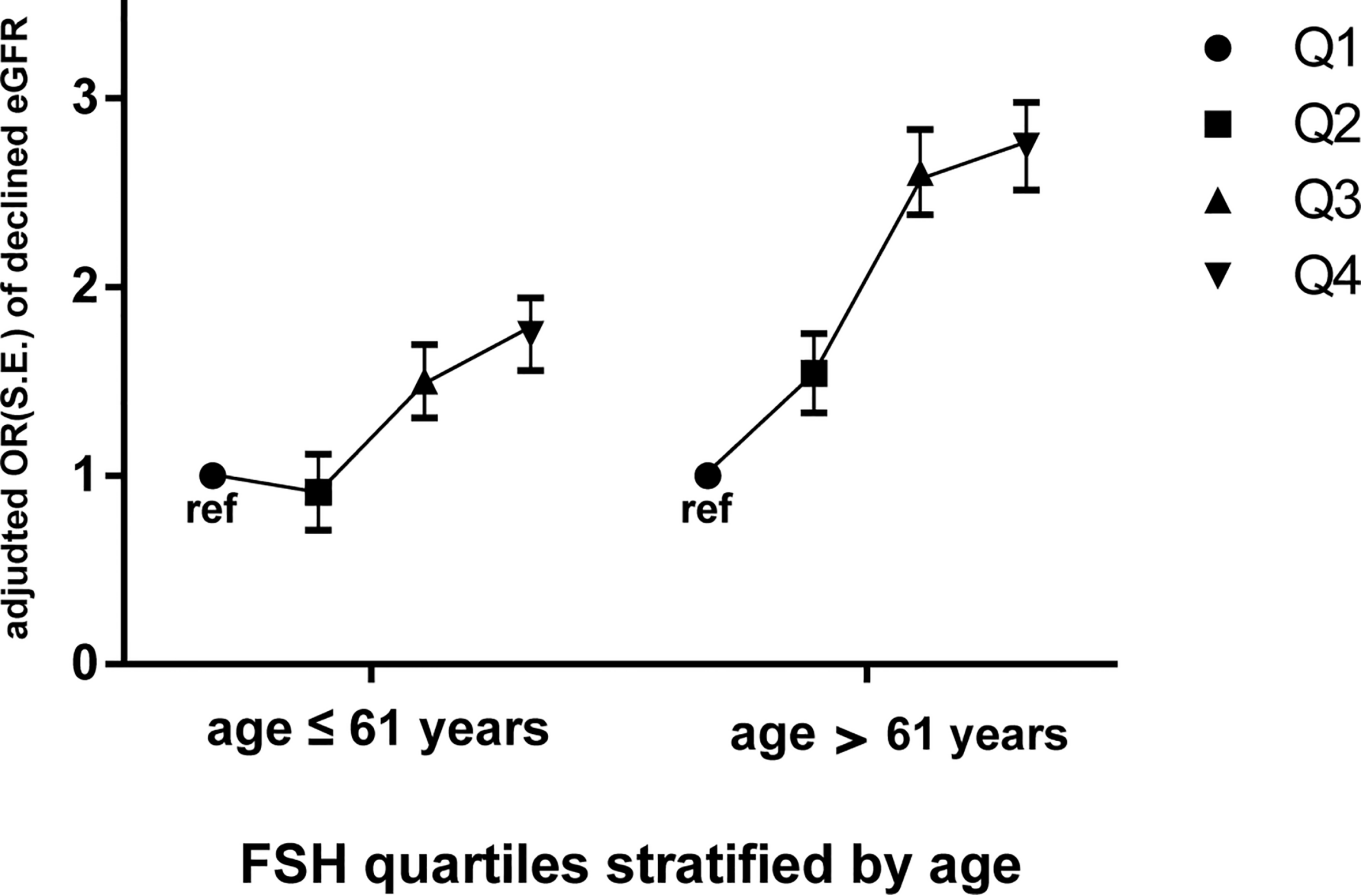
Odds ratios (ORs) of declined eGFR with increasing FSH quartiles (expressed as Q1, Q2, Q3, and Q4) in postmenopausal women stratified by median age (61 years). Dependent variable: declined eGFR; independent variable: FSH quartiles (Q1 as ref). Data are expressed as adjusted odds ratio (OR), standard error (S.E.). Multivariate logistic stepwise regression model: adjusted for age, years since menopause, LH, E2, BMI, dyslipidaemia, diabetes, hypertension, smoking, drinking. FSH, follicle-stimulating hormone; eGFR, estimated glomerular filtration rate; declined eGFR, eGFR<90 mL/min/1.73 m^2^.

We stratified the post-menopausal women into two equal-sized groups (n=1270 each) according to median age (≤61 and >61 years) and conducted a multivariate logistic stepwise regression analysis for each group. After adjusting for age, years since menopause, LH, E2, BMI, dyslipidaemia, diabetes, hypertension, smoking, and drinking, the ORs of declined eGFR increased across FSH quartiles in both age groups; moreover, they were higher for each FSH quartile in the older group (OR=1.461 for quartile 2, OR=2.486 for quartile 3, and OR=2.530 for quartile 4 vs quartile 1) than for the corresponding quartile in the younger group (OR=0.925 for quartile 2, OR=1.498 for quartile 3, and OR=1.755 for quartile 4 vs quartile 1) (**Figure 3** and **Table 5**).

**Table 5:** Logistic stepwise regression of FSH quartiles for the presence of declined eGFR in postmenopausal women stratified by median age.

	B	S.E.	OR (95% CI)	P value
Age ≤ 61 years				
FSH quartile 1			1 (ref)	
FSH quartile 2	-0.093	0.201	0.911 (0.614–1.350)	0.642
FSH quartile 3	0.405	0.194	1.500 (1.026–2.192)	0.036
FSH quartile 4	0.559	0.193	1.748 (1.197–2.554)	0.004
Age > 61 years				
FSH quartile 1			1 (ref)	
FSH quartile 2	0.449	0.211	1.567 (1.037-2.368)	0.033
FSH quartile 3	0.984	0.228	2.676 (1.711-4.183)	<0.001
FSH quartile 4	1.032	0.232	2.807 (1.782-4.421)	<0.001

Stratification variable: age; dependent variable: declined eGFR; independent variable: FSH quartiles; data are expressed as coefficient (B), standard error (S.E.), adjusted odds ratio (OR), 95% confidence interval (CI), and significance (P value). Multivariate model: adjusted for age, years since menopause, LH, E2, BMI, dyslipidaemia (yes=1, no=0): high TC, high TG, high LDL-C, high LDL-C, high FFA, low HDL-C; diabetes (yes=1, no=0), hypertension (yes=1, no=0), smoking (yes=1, no=0), drinking (yes=1, no=0).

FSH, follicle-stimulating hormone; LH, luteinizing hormone; eGFR, estimated glomerular filtration rate; declined eGFR, eGFR<90 ml/min/1.73 m^2^.

## Discussion

Few studies have investigated the association between serum FSH level and renal function in women in the context of menopause. In this study, we found that a high level of circulating FSH was an independent risk factor for renal dysfunction in post-menopausal women and that the adverse impact of FSH was greater in older women (>61 years).

Menopause—a word originating from the Latin term *menopausis* (*meno*=month, *pausis*=pause)—is characterized by amenorrhea for at least 12 months, accompanied by decreased estrogen and increased FSH concentrations as a result of ovarian failure (8). There is increasing evidence that peri- and post-menopausal women have an elevated risk of developing CKD (23–25), suggesting that menopause accelerates the progression of kidney damage (26), although the underlying mechanisms are not fully understood. The decline in estrogen level associated with menopause likely contributes to renal dysfunction in women, as estrogen has been shown to attenuate kidney injury (27) whereas this protective effect was lost with the onset of menopause (2). Post-menopausal status is also associated with an increased risk of metabolic syndrome (MS) (28), which is characterized by abdominal obesity, dyslipidaemia, hypertension, and diabetes, etc. MS can progress to CKD (29) and has been reported as an independent risk factor for the disease (27, 30, 31) particularly in post-menopausal women, even after adjusting for vital confounders (32).

Our results also showed that an increase in circulating FSH level adversely impacted renal function in post-menopausal women. We first compared sex hormone levels and renal function indices among women who were grouped according to menopausal status, and found that a decline in renal function in post-menopausal women was associated with an increased prevalence of renal dysfunction. To determine whether increased FSH levels had an independent association on renal function, post-menopausal women were classified into FSH quartiles and a multivariate logistic regression analysis was carried out with adjustment for potential confounders reported in previous studies. We found that high circulating FSH level was independently associated with an increased risk for renal dysfunction. On the other hand, E2 level was extremely low and age showed no significant trend with increasing FSH quartile. Therefore, this was an ideal group in which to analyze the independent association of FSH on renal function excluding the effects of estrogen and age. Previous studies have shown that FSH can affect the metabolism of non-gonadal organs such as liver (16, 17), adipose tissue (33), and bone (15, 34–36), which is supported by our findings. Moreover, FSH receptor is expressed in kidney tissue (37, 38) and FSH was shown to promote renal tubulointerstitial fibrosis in aging women *via* the protein kinase B (AKT)/glycogen synthase kinase (GSK)‐3β/β‐catenin pathway (38).

Aging accelerates the decline in renal function (22). To investigate the influence of age on the association between FSH and renal dysfunction after menopause, we stratified the post-menopausal women by median age. The older group showed a higher risk of declined eGFR for each FSH quartile, indicating that aging aggravated the adverse impact of FSH on renal function in these women.

The strength of our study was its relative novelty because few studies had investigated the association between FSH levels and renal function in terms of menopause. To best of our knowledge, only Kun Zhang et al. reported the effect of FSH on renal function in aging women. Other strengths included the relatively large sample size of our study and application of various statistical methods, which made more credible results. However, this study had several unsatisfactory results. For example, the prevalence of declined eGFR in post-menopausal women was the same in FSH quartiles 3 and 4 (both 59%) and the prevalence of CKD was the same in FSH quartiles 2 and 3 (both 1.9%), which were not the expected trends. These may be attributed to the characteristics of this single population and an imprecise definition of declined and CKD. Also, there were some limitations in this study. Firstly, because the subjects were from a single population the majority of whom had normal renal function, the overall prevalence of CKD in post-menopausal women was low (1.9%), as was the prevalence in FSH quartiles among these women (0.3–3.3%). Another factor explaining the low prevalence of CKD in our study was that it was defined solely as an eGFR <60 ml/min/1.73 m^2^ without considering elevated urinary protein levels, which precedes the decline in eGFR and is an important criterion for the disease. As a result, the OR of CKD was approximately 10-fold higher with a large 95% CI in the highest FSH quartile compared to the lowest quartile, which undermines the validity of these data. Therefore, additional epidemiologic studies in women with poor renal function may be necessary to confirm the relationship between FSH level and CKD risk. Secondly, while the large sample size of our study increased the reliability of our findings and permitted a stratified analysis, a statistically significant result (eg, the decrease in eGFR and increase in Scr from the lowest to highest FSH quartiles by 3.67 ml/min/1.73 m^2^ and 3.57 μmol/l, respectively) may not be clinically significant; therefore, the results of this study must be interpreted with a certain degree of caution.

In summary, high circulating FSH level was associated with renal dysfunction in post-menopausal women. Even with adjustments for possible confounders, FSH was an independent risk factor for renal dysfunction in this group. Our data also suggested that aging may aggravate the association of high FSH levels with reduced renal function in post-menopausal women. estrogen-based hormone replacement therapy (HRT) can delay CKD progression and some studies have demonstrated improved eGFR in women taking estrogen (13); however, an increased risk of breast cancer and adverse effects on the cardiovascular system following HRT have also been reported (26, 39, 40). The findings of our study provide insight into the pathophysiology of renal dysfunction in women after menopause, which was interpreted as deleterious effects of increased circulating FSH level and suggest that therapeutic strategies that reduce FSH levels can be helpful in preventing renal dysfunction in menopausal women.

## Data Availability Statement

The datasets presented in this article are not readily available because the data are not publicly available due to privacy or ethical restrictions. Requests to access the datasets should be directed to QL (18366117208@163.com&).

## Ethics Statement

The studies involving human participants were reviewed and approved by Ruijin Hospital Ethics Committee of Shanghai JiaoTong University School of Medicine. The patients/participants provided their written informed consent to participate in this study. Written informed consent was obtained from the individual(s) for the publication of any potentially identifiable images or data included in this article.

## Author Contributions

JZ and QL are responsible for the research idea and study design. DZ, HL, and FZ are responsible for data acquisition. JL, YW, ZW, and QG are responsible for data cleansing. QL, QG, MZ, and LG are responsible for data analysis and statistical analysis. All authors contributed to the article and approved the submitted version.

## Funding

This work was supported by National Key Research and Development Program of China (grant nos. 2017YFC1309800 and 2017YFC0909600).

## Conflict of Interest

The authors declare that the research was conducted in the absence of any commercial or financial relationships that could be construed as a potential conflict of interest.
