# Corrigendum: High Circulating Follicle-Stimulating Hormone Level Is a Potential Risk Factor for Renal Dysfunction in Post-Menopausal Women

**DOI:** 10.3389/fendo.2021.710836

**Published:** 2021-08-31

**Authors:** Qihang Li, Dongmei Zheng, Haiyan Lin, Fang Zhong, Jing Liu, Yafei Wu, Zhixiang Wang, Qingbo Guan, Meng Zhao, Ling Gao, Jiajun Zhao

**Affiliations:** ^1^Department of Endocrinology, Shandong Provincial Hospital, Cheeloo College of Medicine, Shandong University, Jinan, China; ^2^Shandong Clinical Medical Center of Endocrinology and Metabolism, Shandong Academy of Clinical Medicine, Jinan, China; ^3^Institute of Endocrinology and Metabolism, Shandong Academy of Clinical Medicine, Jinan, China; ^4^Department of Endocrinology, Shandong Provincial Hospital Affiliated to Shandong First Medical University, Jinan, China; ^5^Health Management Center, Shandong Provincial Hospital Affiliated to Shandong First Medical University, Jinan, China; ^6^Department of Scientific Center, Shandong Provincial Hospital Affiliated to Shandong First Medical University, Jinan, China

**Keywords:** FSH, menopause, eGFR, CKD, renal dysfunction, aging

In the original article, there was a mistake in **Supplementary Table 2D** as published. The **Supplementary Table 2D** was used to show the association between FSH quartiles and the presence of renal dysfunction in postmenopausal women with diabetes by multivariate logistic regression. Due to our fault, the content of the table was mistakenly inserted as the total data in postmenopausal women (the correct data should be in postmenopausal women with diabetes). The corrected **Supplementary Table 2D** appears below. The authors apologize for this error and state that this does not change the scientific conclusions of the article in any way. The original article has been updated.

**Supplementary Table 2a:** Comparisons among groups according to FSH quartiles in postmenopausal women without diabetes.

FSH levels (mIU/mL)	FSH quartile 1	FSH quartile 2	FSH quartile 3	FSH quartile 4	P value	P for trend
eGFR (ml/min/1.73 m^2^)	91.38±9.79	89.83±9.88	88.67±10.28a	87.93±12.02ab	<0.001	<0.001
Declined eGFR, n (%)	201 (37.9%)	238 (45.0%)	274 (51.6%)	273 (51.8%)	<0.001	<0.001
CKD, n (%)	1 (0.2%)	6 (1.1%)	11 (2.1%)	12 (2.3%)	0.015	0.002
Scr (μml/L )	62.57±7.01	63.15±7.33	64.40±7.70ab	65.81±10.96ab	<0.001	<0.001
UA (μml/L)	305±78	302±77	294±73	293±74a	0.017	0.002

All data are expressed as mean ± standard deviation, number (percentage), and significance (P value and P for trend). Scr, serum creatinine; UA, uric acid; eGFR, estimated glomerular filtration rate; declined eGFR, eGFR<90 mL/min/1.73 m^2^; CKD, chronic kidney diseases.

a, compared with FSH quartile1 (P < 0.05).

b, compared with FSH quartile2 (P < 0.05).

c, compared with FSH quartile3 (P < 0.05).

**Supplemetary Table 2b:** Multivariate stepwise logistic regression of FSH quartiles for the presence of renal dysfunction in postmenopausal women without diabetes.

	B	S.E.	OR (95% CI)	P value
Declined eGFR				
FSH quartile 1			1 (ref)	
FSH quartile 2	0.166	0.162	1.181(0.860-1.621)	0.305
FSH quartile 3	0.755	0.164	2.128(1.544-2.935)	<0.001
FSH quartile 4	0.834	0.164	2.303(1.671-3.173)	<0.001

Dependent variable: declined eGFR; independent variable: FSH quartiles; data are expressed as coefficient (B), standard error (S.E.), adjusted odds ratio (OR), 95% confidence interval (CI), and significance (P value). Multivariate model: adjusted for age, years since menopause, LH, E2, BMI, dyslipidaemia (yes=1, no=0): high TC, high TG, high LDL-C, high LDL-C, high FFA, low HDL-C; diabetes (yes=1, no=0), hypertension (yes=1, no=0), smoking (yes=1, no=0), drinking (yes=1, no=0).

FSH, follicle-stimulating hormone; LH, luteinizing hormone; eGFR, estimated glomerular filtration rate; declined eGFR, eGFR<90 ml/min/1.73 m^2^.

**Supplementary Table 2c:** Comparisons among groups according to FSH quartiles in postmenopausal women with diabetes.

FSH levels (mIU/mL)	FSH quartile 1	FSH quartile 2	FSH quartile 3	FSH quartile 4	P value	P for trend
eGFR (ml/min/1.73 m^2^)	87.28±9.33	84.74±12.30	84.91±12.03	81.76±16.22a	0.020	0.003
Declined eGFR, n (%)	58 (55.2%)	65 (61.9%)	69 (65.7%)	67 (64.4%)	0.407	0.140
CKD, n (%)	1 (1.0%)	3 (2.9%)	4 (3.8%)	9 (8.7%)	0.034	0.005
Scr (μml/L )	65.15±6.98	66.98±10.79	67.03±14.15	70.93±20.16a	0.023	0.004
UA (μml/L)	306±81	301±82	288±72	301±90	0.425	0.448

All data are expressed as mean ± standard deviation, number (percentage), and significance P value and (P for trend). Scr, serum creatinine; UA, uric acid; eGFR, estimated glomerular filtration rate; declined eGFR, eGFR<90 mL/min/1.73 m^2^; CKD, chronic kidney diseases.

a, compared with FSH quartile1 (P < 0.05).

b, compared with FSH quartile2 (P < 0.05).

c, compared with FSH quartile3 (P < 0.05).

**Supplementary Table 2d:** Multivariate logistic regression of FSH quartiles for the presence of renal dysfunction in postmenopausal women with diabetes.

	B	S.E.	OR (95% CI)	P value
Declined eGFR				
FSH quartile 1			1 (ref)	
FSH quartile 2	-0.003	0.341	0.997(0.511-1.946)	0.994
FSH quartile 3	0.130	0.351	1.138(0.572-2.267)	0.712
FSH quartile 4	0.606	0.408	1.833(0.825-4.077)	0.137

Dependent variable: declined eGFR; independent variable: FSH quartiles; data are expressed as coefficient (B), standard error (S.E.), adjusted odds ratio (OR), 95% confidence interval (CI), and significance (P value). Multivariate model: adjusted for age, years since menopause, LH, E2, BMI, dyslipidaemia (yes=1, no=0): high TC, high TG, high LDL-C, high LDL-C, high FFA, low HDL-C; diabetes (yes=1, no=0), hypertension (yes=1, no=0), smoking (yes=1, no=0), drinking yes=1, no=0).

FSH, follicle-stimulating hormone; LH, luteinizing hormone; eGFR, estimated glomerular filtration rate; declined eGFR, eGFR<90 ml/min/1.73 m^2^.

In the original article, there was an error. The definition of pre-menopause and peri-menopause in section of *Materials and Methods* was not accurate enough.

A correction has been made to *Materials and Methods, Definitions of Study Outcomes, 1:*

Menopausal status was determined based on responses to a self-report questionnaire regarding menstrual history or amenorrhea. Pre-menopause was defined as the presence of menses within the past 3 months. Peri-menopause was defined as the presence of menses within the past 3 months with menstrual irregularity in the year preceding the questionnaire, or 3–11 months of amenorrhea. We selected subjects with 3–11 months of amenorrhea as peri-menopause because their E2 levels were similar to those in post-menopause. Post-menopause was defined as the cessation of menstruation for a minimum of 12 months (17, 19). Renal dysfunction was defined as declined estimated glomerular filtration rate (eGFR<90 ml/min/1.73 m^2^) or CKD (eGFR<60 ml/min/1.73 m^2^). Dyslipidaemia was defined as follows: 1) high total cholesterol (≥ 6.22 mmol/l); 2) high triglyceride (≥1.70 mmol/l); 3) high low-density lipoprotein cholesterol (≥ 4.14 mmol/l); 4) high free fatty acids (≥ 0.9 mmol/l); and 5) low high-density lipoprotein cholesterol <1.30 mmol/l) (20). Hypertension and diabetes were diagnosed based on self-reported previous diagnosis, or were defined as systolic blood pressure ≥130 mmHg or diastolic blood pressure ≥ 85 mmHg for hypertension (21) and fasting plasma glucose ≥7.0 mmol/l or post-prandial 2-h plasma glucose ≥11.1 mmol/l for diabetes (we chooe the former). Never smoking or drinking was assigned a value of 0; otherwise, it was assigned a value of 1.

The authors apologize for these errors and state that this does not change the scientific conclusions of the article in any way. The original article has been updated.

## Publisher’s Note

All claims expressed in this article are solely those to the authors and do not necessarily represent those of their affiliated organization, or those of the publisher, the editors and reviewers. Any product that may be evaluated in this article, or claim that may be made by its manufacturer, is not guaranteed or endorsed by the publisher.

