# Supplementary material for: High Circulating Follicle-Stimulating Hormone Level Is a Potential Risk Factor for Renal Dysfunction in Post-Menopausal Women
Source: Front Endocrinol (Lausanne). 2021 Apr 1;12:627903. doi: 10.3389/fendo.2021.627903 (PMC8047631; doi:10.3389/fendo.2021.627903)
Supplement: Supplementary file 1 [file DataSheet_1.pdf]

**Supplementary table 1** Comparisons of renal function among groups of LH quartiles in postmenopausal women

| LH level(mIU/ml) | Quartile1 (~24.65) | Quartile2 (24.65-31.38) | Quartile3 (31.38-39.81) | Quartile4 (39.81~) | P value | P for trend |
|---|---|---|---|---|---|---|
| Age (years) | 62.11±7.034 | 61.79±7.21 | 60.92±6.59[a,b] | 58.97±7.01[a,b,c] | <0.001 | <0.001 |
| Scr(μml/L ) | 63.65±7.24 | 63.60±7.04 | 64.88±9.84[a,b] | 66.18±13.20[a,b,c] | <0.001 | <0.001 |
| UA(μml/L) | 300±77 | 299±76 | 300±77 | 294±74 | 0.484 | 0.210 |
| eGFR(ml/min per 1.73 m$^2$) | 88.80±10.079 | 89.23±10.16 | 88.44±11.44 | 88.36±13.25 | 0.505 | 0.294 |
| Declined eGFR,n (%) | 306(48.3%) | 307(48.7%) | 321(50.8%) | 308(48.8%) | 0.821 | 0.696 |
| CKD,n (%) | 5(0.8%) | 6(1.0%) | 13(2.1%) | 23(3.6%) | <0.001 | <0.001 |

All data are expressed as mean ± standard deviation, number (percentage), and significance (P value and P for trend). Scr, serum creatinine; UA, uric acid; eGFR, estimated glomerular filtration rate; declined eGFR, eGFR＜90 mL/min/1.73 m$^2$; CKD, chronic kidney diseases

a, compared with LH quartile1 (P < 0.05)

b, compared with LH quartile2 (P < 0.05)

c, compared with LH quartile3 (P < 0.05)

**Supplementary table 2**

**Supplementary table 2a** Comparisons among groups according to FSH quartiles in postmenopausal women without diabetes

| FSH levels （mIU/mL) | FSH quartile 1 | FSH quartile 2 | FSH quartile 3 | FSH quartile 4 | P value | P for trend |
|---|---|---|---|---|---|---|
| eGFR (ml/min/1.73 m$^2$) | 91.38±9.79 | 89.83±9.88 | 88.67±10.28a | 87.93±12.02ab | ＜0.001 | ＜0.001 |
| Declined eGFR, n (%) | 201（37.9%） | 238（45.0%） | 274（51.6%） | 273（51.8%） | ＜0.001 | ＜0.001 |
| CKD, n (%) | 1（0.2%） | 6（1.1%） | 11（2.1%） | 12（2.3%） | 0.015 | 0.002 |
| Scr (μml/L ) | 62.57±7.01 | 63.15±7.33 | 64.40±7.70ab | 65.81±10.96ab | ＜0.001 | ＜0.001 |
| UA (μml/L) | 305±78 | 302±77 | 294±73 | 293±74a | 0.017 | 0.002 |

All data are expressed as mean ± standard deviation, number (percentage), and significance (P value and P for trend). Scr, serum creatinine; UA, uric acid; eGFR, estimated glomerular filtration rate; declined eGFR, eGFR＜90 mL/min/1.73 m$^2$; CKD, chronic kidney diseases

a, compared with FSH quartile1 (P < 0.05)

b, compared with FSH quartile2 (P < 0.05)

c, compared with FSH quartile3 (P < 0.05)

**Supplementary table 2b** Multivariate stepwise logistic regression of FSH quartiles for the presence of renal dysfunction in postmenopausal women without diabetes

| | B | S.E. | OR (95% CI) | P value |
|---|---|---|---|---|
| Declined eGFR | | | | |
| FSH quartile 1 | | | 1 (ref) | |
| FSH quartile 2 | 0.166 | 0.162 | 1.181(0.860-1.621) | 0.305 |
| FSH quartile 3 | 0.755 | 0.164 | 2.128(1.544-2.935) | ＜0.001 |
| FSH quartile 4 | 0.834 | 0.164 | 2.303(1.671-3.173) | ＜0.001 |

Dependent variable: declined eGFR ; independent variable: FSH quartiles; data are expressed as coefficient (B), standard error (S.E.), adjusted odds ratio (OR), 95% confidence interval (CI), and significance (P value). Multivariate model: adjusted for age, years since

menopause, LH, E2, BMI, dyslipidaemia (yes=1, no=0): high TC, high TG, high LDL-C, high LDL-C, high FFA, low HDL-C; diabetes (yes=1, no=0), hypertension (yes=1, no=0), smoking (yes=1, no=0), drinking (yes=1, no=0)

FSH, follicle-stimulating hormone; LH, luteinizing hormone; eGFR, estimated glomerular filtration rate; declined eGFR, eGFR<90 ml/min/1.73 $m^2$

**Supplementary table 2c** Comparisons among groups according to FSH quartiles in postmenopausal women with diabetes

| FSH levels （mIU/mL) | FSH quartile 1 | FSH quartile 2 | FSH quartile 3 | FSH quartile 4 | P value | P for trend |
|---|---|---|---|---|---|---|
| eGFR (ml/min/1.73 $m^2$) | 87.28±9.33 | 84.74±12.30 | 84.91±12.03 | 81.76±16.22a | 0.020 | 0.003 |
| Declined eGFR, n (%) | 58（55.2%） | 65（61.9%） | 69（65.7%） | 67（64.4%） | 0.407 | 0.140 |
| CKD, n (%) | 1（1.0%） | 3（2.9%） | 4（3.8%） | 9（8.7%） | 0.034 | 0.005 |
| Scr (μml/L ) | 65.15±6.98 | 66.98±10.79 | 67.03±14.15 | 70.93±20.16a | 0.023 | 0.004 |
| UA (μml/L) | 306±81 | 301±82 | 288±72 | 301±90 | 0.425 | 0.448 |

All data are expressed as mean ± standard deviation, number (percentage), and significance (P value and P for trend). Scr, serum creatinine; UA, uric acid; eGFR, estimated glomerular filtration rate; declined eGFR, eGFR＜90 mL/min/1.73 $m^2$; CKD, chronic kidney diseases

a, compared with FSH quartile1 (P < 0.05)

b, compared with FSH quartile2 (P < 0.05)

c, compared with FSH quartile3 (P < 0.05)

**Supplementary table 2d** Multivariate logistic regression of FSH quartiles for the presence of renal dysfunction in postmenopausal women with diabetes

| | B | S.E. | OR (95% CI) | P value |
|---|---|---|---|---|
| Declined eGFR | | | | |
| FSH quartile 1 | | | 1 (ref) | |
| FSH quartile 2 | -0.003 | 0.341 | 0.997(0.511-1.946) | 0.994 |
| FSH quartile 3 | 0.130 | 0.351 | 1.138(0.572-2.267) | 0.712 |
| FSH quartile 4 | 0.606 | 0.408 | 1.833(0.825-4.077) | 0.137 |

Dependent variable: declined eGFR ; independent variable: FSH quartiles; data are expressed as coefficient (B), standard error (S.E.), adjusted odds ratio (OR), 95% confidence interval (CI), and significance (P value). Multivariate model: adjusted for age, years since menopause, LH, E2, BMI, dyslipidaemia (yes=1, no=0): high TC, high TG, high LDL-C, high LDL-C, high FFA, low HDL-C; diabetes (yes=1, no=0), hypertension (yes=1, no=0), smoking (yes=1, no=0), drinking (yes=1, no=0)

FSH, follicle-stimulating hormone; LH, luteinizing hormone; eGFR, estimated glomerular filtration rate; declined eGFR, eGFR<90 ml/min/1.73 $m^2$
